# Hysteroscopy among Patients Attending the Outpatient Department of Gynaecology in a Tertiary Care Centre: A Descriptive Cross-sectional Study

**DOI:** 10.31729/jnma.8063

**Published:** 2023-03-31

**Authors:** Asmita Ghimire, Sailaja Ghimire, Asmita Shrestha, Samriddha Raj Pant, Nilam Subedi, Poonam Koirala, Padam Raj Pant

**Affiliations:** 1 Department of Obstetrics and Gynecology, Tribhuvan University Teaching Hospital, Maharajgunj, Kathmandu, Nepal; 2 Department of Obstetrics and Gynecology, Bhim District Hospital, Bhairahawa, Rupandehi, Nepal; 3 Department of Obstetrics and Gynecology, Grande International Hospital, Dhapasi, Kathmandu, Nepal; 4 Maharajgunj Medical Campus, Maharajgunj, Kathmandu, Nepal

**Keywords:** *hysteroscopy*, *infertility*, *leiomyoma*, *polyps*

## Abstract

**Introduction:**

Hysteroscopy is a procedure used widely for diagnostic and therapeutic purposes. Hysteroscopy enables visualisation of the endometrial cavity and if possible treatment in the same setting avoiding invasive procedure. The objective of the study was to find out the prevalence of hysteroscopy among gynaecological patients attending the outpatient department of Obstetrics and Gynaecology in a tertiary care centre.

**Methods:**

A descriptive cross-sectional study was done among gynaecological patients from 1 January 2016 to 1 January 2020 visiting the outpatient Department of Obstetrics and Gynaecology in a tertiary care centre after ethical approval from the Institutional Review Committee (Registration number: 029/2021). Convenience sampling was used. Data regarding demographic parameters, hysteroscopy findings, procedures performed, histopathological findings and complications were retrieved from the electronic database of the hospital. Point estimate and 95% Confidence Interval were calculated.

**Results:**

Among 319 gynaecological patients, hysteroscopy was done in 72 (22.57%) (17.98-27.16, 95% Confidence Interval) patients.

**Conclusions:**

The prevalence of hysteroscopy among gynaecological patients was higher to the studies done in similar settings.

## INTRODUCTION

Hysteroscopy is a diagnostic and operative procedure used worldwide for intrauterine pathology.^1^ Gradually over the years, it has become a tool of choice and it is replacing dilatation and curettage in treating conditions like abnormal uterine bleeding.^2,3^ Hysteroscopy enables visualisation of the endometrial cavity and if possible treatment in the same setting avoiding the need for an invasive procedure.

It helps in the complete visualization of endometrium revealing normal and abnormal intrauterine pathology. Even cases of missing Intrauterine Contraceptive devices (IUCD), synechiae removal and infertility can be tackled with ease.^4^

The objective of the study was to find out the prevalence of hysteroscopy among gynaecological patients attending the outpatient department of Obstetrics and Gynaecology in a tertiary care centre.

## METHODS

A descriptive cross-sectional study was done for a duration of five years from 1 January 2016 to 1 January 2020 among gynaecological patients visiting the outpatient Department of Obstetrics and Gynaecology of Grande International Hospital (GIH), Dhapasi, Kathmandu, Nepal after taking ethical approval from the Institutional Review Committee of GIH (Registration number: 029/2021). All patients visiting outpatients Department of Gynaecology during study period were included in the study. Patients with irregular menstrual cycles and with continuous per vaginal bleeding were excluded. Convenience sampling was used. The sample size was calculated using the following formula:


$$\begin{array}{ll} \text{n} & ={\text{Z}}^{2}\times \frac{\text{p}\times \text{q}}{{\text{e}}^{\text{2}}}\\ & =1.96^{2}\times \frac{0.5\times 0.5}{0.06^{2}}\\ & =267 \end{array}$$

Where,

n = minimum required sample size
Z = 1.96 at 95% of Confidence Interval (CI)
p = prevalence taken as 50% for maximum sample size
q= 1-p
e = margin of error, 6%

The calculated sample size was 267. However, a total of 319 patients were included.

Each of the patients were given 400 mcg misoprostol per vaginally four hours prior to the procedure. The surgical instrument used was a rigid storz hysteroscope with 0° or 30° lenses. Cutting loop, coagulating electrode, biopsy forceps and scissors were commonly used.^5^

The media used was normal saline. All examinations were performed by a single gynaecologist with specialized training in gynaecological endoscopy experience of more than 15 years. The standard sequencing of visualization of the endometrial cavity was maintained. The order followed was a visualisation of the ectocervix, endocervical canal, walls of the uterus, uterine fundus, Ostia and finally the endometrial characteristics. Hysteroscopic findings were thus documented and video-recorded. As per need, diagnostic and therapeutic management was done. Obtained tissue was sent for histopathological examination. Demographic parameters of the patients like age, parity, abortion, menopause, symptoms, and diagnosis along with hysteroscopic and histopathological findings and procedures performed were retrieved from the electronic database maintained in the hospital record. Care was taken to maintain patient confidentiality.

Data were analysed using IBM SPSS Statistics version 22.0. Point estimate and 95% CI were calculated.

## RESULTS

Among 319 gynaecological patients, hysteroscopy was done in 72 (22.57%) (17.98-27.16, 95% CI) patients. From them, 60 (83.33%) of those patients had endometrial tissue samples sent for histopathology. Among the remaining 12 cases, 5 (41.67%) patients had synechiae release, 3 (25%) had septum resections, 3 (25%) had IUCD removal and 1 (8.33%) had foreign body removal. A total of 33 (45.83%) patients were in the age group of 31-40 (Table 1).

**Table 1:** Demographic parameters (n = 72).

Parameters	Group	n (%)
Age group (years)	<20	2 (2.78)
	21-30	25 (34.72)
	31-40	33 (45.83)
	41-50	9 (12.50)
	51-60	2 (2.78)
	>61	1 (1.39)
Parity	0	33 (45.83)
	1	10 (13.89)
	2	22 (30.56)
	3	6 (8.33)
	4	1 (1.39)
Number of abortions	0	44 (61.11)
	1	14 (19.44)
	2	8 (11.11)
	3	5 (6.95)
	6	1 (1.39)
Menopausal status	Premenopause	67 (93.05)
	Menopause	5 (6.95)

**Figure 1:**
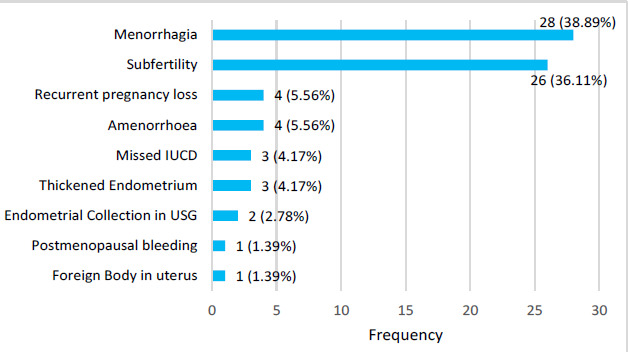
Bar diagram showing the number of patients with different symptoms among patients undergoing hysteroscopy (n= 72).

Excessive vaginal bleeding during menstruation was seen in 28 (38.88%) as common presenting complaint followed by inability to conceive 26 (36.11%) (Figure 1).

**Figure 2:**
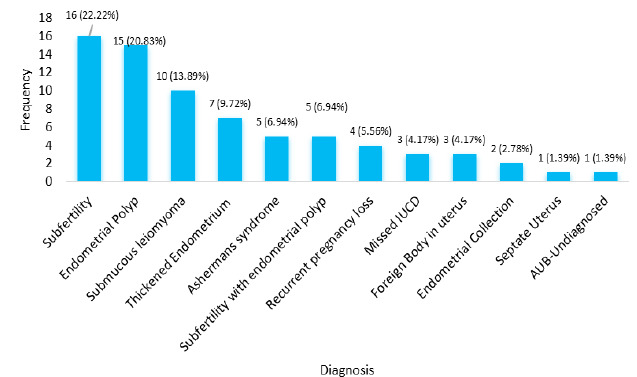
Bar diagram showing the number of patients with different diagnoses after hysteroscopy (n= 72).

The commonest diagnosis at presentation was subfertility 16 (22.22%) followed by endometrial polyp 15 (20.83%) with both diagnoses together present in 5 (6.94%) (Figure 2).

**Figure 3:**
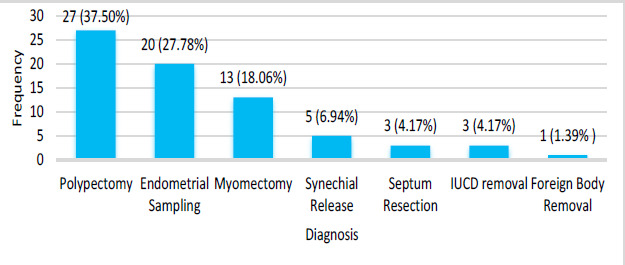
Bar diagram showing the number of patients with different hysteroscopic procedures performed (n= 72).

Total of 27 (37.50%) patients underwent polypectomy and 20 (27.78%) underwent endometrial sampling (Figure 3).

The hysteroscopy findings of endometrial polyp 31 (43.05%) was confirmed in only 28 (46.67%) cases by histopathological examination (Table 2).

**Table 2:** Histopathological findings (n= 60).

Findings	n (%)
Endometrial polyp	28 (46.67)
Submucosal leiomyoma	10 (16.67)
Proliferative phase endometrium	9 (15)
Secretory phase endometrium	11 (18.33))
Uterine synechiae	-
Uterine septum	-
IUCD embedded in uterine cavity	-
Foreign body	-
Endometrial hyperplasia with focal atypia	2 (3.33)

## DISCUSSION

Hysteroscopy enables visualisation of the endometrial cavity and if possible treatment in the same setting. It is a minimally invasive procedure used worldwide. The American College of Obstetricians and Gynecologists and the American Association of Gynecologic Laparoscopists recommend the use of hysteroscopy for the diagnosis and treatment of intrauterine pathology.^4^ The recommendation of using 400 mcg per vaginal misoprostol 4 hours prior to the procedure was followed in this study.

The present study showed the prevalence of hysteroscopy to be higher than in other studies.^5^ This could be because the present study was done in a centre where there were more infertile cases approaching and this centre was among the few in the country where hysteroscopy is performed.

In the present study, the most common presenting complaint was excessive vaginal bleeding 38.88% followed by inability to conceive 36.11%. Similarly, in a study done abnormal uterine bleeding 32.5% was the presenting complaint.^6^

Endometrial polyp 43.05% was the commonest hysteroscopy finding in the present study similar to the study done in Libya was 53.6%.^5^ However, it was contrary to the study done in India^7^ where most patients presented with proliferative endometrium 34%. This could be because in their study they had included patients of 40 years and above with abnormal uterine bleeding only, however, we had patients with ages ranging from 20-65 years with various symptoms.

There were five cases of uterine synechiae in which adhesiolysis was done after which three cases were able to conceive. A study done in Linkou^8^ that carried adhesiolysis in 85 females with Asherman's syndrome had also shown excellent results. Uterine septum resection was done in three cases with conception occurring within six months in two cases. The present study had 13 patients 43% of who conceived postsurgery mostly through in vitro fertilization. A study done in Belgium^9^ also showed a higher pregnancy rate of 63% after the removal of endometrial polyp prior to intrauterine insemination. Higher fertility rates thus usually occur in women seeking fertility treatment after hysteroscopic removal of an endometrial polyp, submucosal leiomyoma, uterine septum and intrauterine adhesions.

Three cases of missing IUCD and one case of a foreign body were also successfully removed in the present study. No significant complications occurred except in two cases 2.77% of fluid overload was managed with diuretics. The complication rate was also 2% in a study done in Libya^10^ however, they were of different types of postoperative haemorrhage and perforation. This could be because of more number of cases in their study. Another reason could be the lack of consistency and experience of the surgeons doing hysteroscopy. Similar to the present study, a study done in Iran^5^ also showed complications of fluid overload in only a few cases.

The point of confusion in the case of proliferative and secretory endometrium could be because of the history of intake of hormonal pills. In patients with such an intake of pills, the endometrium shows mixed characteristics.^11^ In the same endometrial cavity, there could be some areas of proliferative and some areas of secretory endometrium. Also, endometrial polyps and submucosal leiomyoma can present in the background of either secretory or proliferative endometrium leading to false results.

The limitation of the study is thus its small sample size. Bigger sample populations with more named patterns on hysteroscopy are required as sometimes one histology may give more than one picture on hysteroscopic analysis. Also since it is a retrospective study, data might have been missed.

## CONCLUSIONS

The prevalence of hysteroscopy among gynaecological patients was higher than in the studies done in similar settings. Hysteroscopy enables visualisation of the endometrial cavity and if possible treatment in the same setting avoiding the need for an invasive procedure.
